# Effect of acetylcholine deficiency on neural oscillation in a brainstem-thalamus-cortex neurocomputational model related with Alzheimer’s disease

**DOI:** 10.1038/s41598-022-19304-3

**Published:** 2022-09-02

**Authors:** Hao Yang, XiaoLi Yang, SiLu Yan, ZhongKui Sun

**Affiliations:** 1 School of Mathematics and Statistics, Shaanxi Normal University, Xi’an, 710062 People’s Republic of China; 2 School of Mathematics and Statistics, Northwestern Polytechnical University, Xi’an, 710072 People’s Republic of China

**Keywords:** Computational biology and bioinformatics, Neuroscience

## Abstract

Previous works imply that involving brainstem in neuropathological studies of Alzheimer’s disease (AD) is of clinically significant. This work constructs a comprehensive neural mass model for cholinergic neuropathogenesis that involves brainstem, thalamus and cortex, wherein how acetylcholine deficiency in AD affects neural oscillation of the model output is systematically explored from the perspective of neurocomputation. By decreasing synapse connectivity parameters in direct cholinergic pathway from brainstem to thalamus or in indirect glutamatergic synapse pathway from cortex to brainstem to mimic the pathological condition of reduced acetylcholine release in patients with AD, the property of neural oscillation in this model is numerically investigated by means of power spectrum in frequency domain and amplitude distribution in time domain. Simulated results demonstrate that decreasing synapse connectivity whether in the direct cholinergic pathway or in the indirect glutamatergic synapse pathway can alter the neural oscillation significantly in three aspects: it induces an obvious decrease of dominant frequency; it leads to a degraded rhythmic activity in the alpha frequency band as well as an enhanced rhythmic activity in the theta frequency band; it results in reduced oscillation amplitude of the model output. These results are agreement with the characteristic of electrophysiological EEG measurement recorded in AD, especially support the hypothesis that cholinergic deficiency is a promising pathophysiological origin of EEG slowing in AD. Our analysis indicates that targeting the cholinergic system may have potential prospects in early diagnosis and treatment of AD.

## Introduction

As we all know, the brain is the largest and most complex structure of the central nervous system, which is closely related to consciousness, memory and other activities^1^. Damage to some brain regions would cause neurological or psychiatric disorders such as epilepsy, Alzheimer’s disease (AD) and autism. AD is a neurodegenerative disease of the nervous system in the elderly. Only when irreversible damage occurs to the brain can AD be detected, so it poses a very serious threat to the health of AD patients^2^.

The cholinergic hypothesis indicates that the cholinergic system in patients with AD is abnormal. Cholinergic action is important to maintain attention and memory^3,4^. Relevant studies have found that there are massive loss of cholinergic neurons and decrease of acetylcholine release in the brain of AD patients^5–8^. In addition, the brainstem contains a large number of cholinergic neurons projecting to the thalamus^9–11^ which can maintain the cortical excitability during wakefulness and rapid eye movement sleep^12,13^. In order examine that the brainstem nuclei are vulnerable to AD-related pathological changes, Parvizi et al.^14^ have performed a study of thioflavin S-stained serial sections in the entire brainstem, in which they have found that the physiological markers of AD, senile plaques and neurofibrillary tangles, are respectively existed in superior colliculus and cholinergic nucleus in 32 AD patients, whereas no changes are seen in the brainstem of 26 normal subjects. The above findings imply that AD may relate with the degeneration of cholinergic neurons in the brainstem, especially the cholinergic pathway from the brainstem to the thalamus may be important to understand the pathogenesis of AD.

There have been considerable laboratory researches, such as neuroimaging, neurochemistry and gene mapping, to identify potential AD marker for early preventative treatments of AD. Electroencephalographic (EEG), for its sensitivity to brain pathology, relative non-invasiveness and ease of measurement, has become a popular neurophysiological technique to study AD. Some typical EEG changes including the reduced dominant frequency (also called peak frequency)^15–17^ and the slowing of EEG signals in AD patients^17–21^ have been revealed. Moreover, in a review of EEG dynamics in AD patients, Jeong^20^ has stated that the pathophysiological origin for EEG slowing in AD may be largely due to acetylcholine deficiency. This statement is also supported by EEG experiment in healthy subjects using scopolamine (a kind of muscarinic receptor antagonist), in which there appears an increase in the delta and theta power, and a decrease in the alpha and beta power after administrating scopolamine^22^.

In order to better understand the mechanism behind the activity of brain neurons, many neurocomputational models have been developed, roughly categorized as detailed model and neural mass model^23–29^. The detailed model is too computationally complicated to analyze the whole behavior in a relatively large brain region. For the neural mass model, each cell population represents a neuronal ensemble of mesoscopic scale, which is lumped together and supposed to share same membrane potential. One of the first neural mass model was proposed first by Wilson and Cowan^30,31^ based on the interaction of one excitatory population and one inhibitory population. Then, Silva et al.^32^ have established an alpha rhythm model in the thalamus to mimic the generation of alpha rhythm. Subsequently, Jansen and Rit^33^ have changed the impulse response function to construct a neural mass model in a single cortical column. Wendling et al.^34^ have included the population of GABA interneurons with fast synaptic kinetics into the Jason and Rit’s model, where they produced realistic multichannel epileptiform EEG signal in the hippocampus. Nowadays, the neural mass model proposed by Jansen and Rit has been frequently extended to coupled neural mass models to simulate the complexity of EEG dynamics in large cortical region^28,29,34–36^. In addition, Bhattacharya et al. have proposed a thalamus-cortex-thalamus (TCT) neural mass model consisting of a thalamic module and a cortical module, in which the synaptic connectivity related to the aberration of the alpha rhythm in the brain resulting from AD is discussed^37^. Recently, Li et al. have improved the TCT model by incorporating the disinhibition property between different inhibitory interneurons in the cortical region and introducing the full relay function of thalamus to the cortical region^26^, which is helpful to understand the neuronal correlates of slowing of the alpha rhythm induced by AD.

Although some neural mass models comprising cortical column, thalamus or hippocampus have been successfully exploited to simulate some specific aspects of brain rhythms and the abnormal brain activity during disease, they have not been used to analyze the underlying brain dynamics in the brainstem associated with AD. In addition, as indicted in the previous work^9,12,14^, in brainstem there are different nuclei involving in many functions, such as controlling homeostasis and emotions, modulating the cognitive functions of cerebral cortex, thus understanding its involvement in AD is clinically significant. Nevertheless, the status of most subcortical structures including the brainstem remains poorly understood during AD compared with the majority of neuropathological studies of AD from the cortical aspect. Some manifestations of AD (e.g. behavioral, affective, and cognitive abnormality) may be interpreted from the perspective of dysfunction in subcortical structure of brainstem^14^. Thus, with a purpose to mimic the EEG dynamics observed in patients of AD, this work extends the neural mass model to build a comprehensive model in the interactive brain structures of brainstem, thalamus and cortex (i.e., a BTC model), in which how abnormal acetylcholine release affects the neural oscillations of the model output is explored.

The following is organized as follows. Firstly, model presentations including how to construct the BTC model and its related model parameters are illustrated in “An enhanced neural mass model: brainstem thalamus cortex model” Section . Then the effect of reduced synapse connectivity resulting from AD on the neural oscillation in this BTC model is systematically studied by numerical simulations in “Main result” Section. Finally, a brief summary of this work is present.

### An enhanced neural mass model: brainstem-thalamus-cortex model

**Figure 1:**
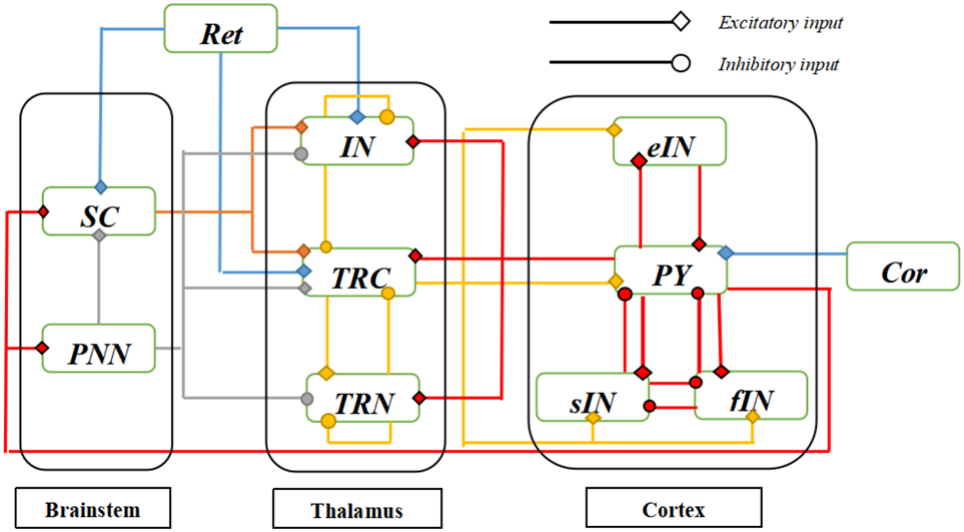
The layout for module structure and synaptic connectivity in the BTC model. The BTC model comprises of three formations: brainstem module, thalamus module and cortex module. In the brainstem module there are two populations of superior colliculus (SC) and pedunculopontine nucleus (PNN). In the thalamus module there are thalamic relay nucleus (TRC), inhibitory interneurons (IN) and thalamic reticular nucleus (TRN). In the cortex module there are pyramidal neurons(PY), excitatory interneurons (eIN), fast inhibitory interneurons (fIN) and slow inhibitory interneurons (sIN). Ret and Cor denote external visual inputs and adjacent cortical inputs respectively. Diamond represents excitatory inputs and circle represents inhibitory inputs.

As described in the Introduction, in addition to the thalamic and cortical aspects, the involvement of brainstem is also of significance to AD^9,12,14^. In this section, based on the neural mass models in cortical and thalamic regions^26,37^, a comprehensive neural mass model in the interactive brain regions of brainstem, thalamus and cortex (BTC) is firstly constructed by newly adding two brainstem neuron populations-superior colliculus and the pedunculopontine nucleus. As shown in Fig. 1, this BTC model is made of a brainstem module, a thalamus module and a cortex module. There are two populations of superior colliculus (SC) and pedunculopontine nucleus (PNN) in the brainstem module, where the PNN population produces excitatory synapse on the SC population, as implied by the work^11^. The thalamus module is modeled by three populations of thalamic relay nucleus (TRC), inhibitory interneurons (IN) and thalamic reticular nucleus (TRN)^26,32,37–39^. The TRC population receives inhibitory inputs from the IN and TRN populations via intrinsic synapse pathway, whereas the TRN population receives excitatory inputs from the TRC population. The IN and TRN populations also receive inhibitory inputs from themselves, respectively. The module of cortex is built upon four populations of pyramidal neurons (PY), excitatory interneurons (eIN), fast inhibitory interneurons (fIN) and slow inhibitory interneurons (sIN)^26,28,29,34,37^. Within this module, the PY population sends excitatory outputs to the sIN and fIN populations, in turn, both the sIN and fIN populations send inhibitory outputs to the PY population. There are reciprocal excitatory outputs between the PY and eIN populations, and there are reciprocal inhibitory outputs between the sIN and fIN populations. For the interconnections between the three modules, as indicated in the studies^9–11,40,41^, there are cholinergic and glutamatergic synapse pathway from the brainstem area to the thalamus area. The thalamic nuclei are modulated by an ascending cholinergic projection from the brainstem via cholinergic neurotransmitter. Depending on different muscarinic receptor subtypes, the PNN population sends exhibitory outputs to the TRC population by means of M1 and M3 receptor subtypes^42^, also it sends inhibitory outputs to the IN and TRN populations by means of M2 receptor subtype^9–11,43^. Meanwhile, the SC population in the brainstem sends excitatory outputs to the TRC and IN populations via glutamatergic neurotransmitter^40,41^. The synaptic interconnections between the thalamus and the cortex modules conform to the classical results as presented in the literatures^26,36,37^, i.e., all the populations of TRC, IN and TRN in the thalamus region receive excitatory inputs from the PY population, on the same time, the TRC population sends excitatory outputs to all the populations of PY, eIN, fIN and sIN in the cortical region. In addition to the ascending synapses from the brainstem to the thalamus and cortex, there is important descending synapse pathway from the cortex to the brainstem in the central nervous system^44,45^. Here, the PY population in the cortex module sends excitatory outputs to the SC and PNN populations in the brainstem module. Other extrinsic sources to the BTC model come from the retinal and nearby cortical formations, i.e., the retinal population sends excitatory outputs to the IN, TRC and SC populations, and the PY population receives excitatory afferent from nearby cortical regions^26,28,29,36,37^.

For convenience, neuron populations in this model are denoted by different lowercase letters $s$, $d$, $i$, $t$, $n$, $h$, $p$, $f$, $l$, $r$ and $c$, which represent populations SC, PNN, IN, TRC, TRN, eIN, PY, fIN, sIN, Ret, Cor, respectively. According to the technique of neural mass model, the kinetic equations governing each population are obtained by performing two mathematical operations. One is that the average membrane potential $V_{a}$ in each population $a$ (here $a$ = $s$, $d$, $i$, $t$, $n$, $h$, $p$, $f$, $l$, $r$, $c$) receiving from all afferent neuronal populations is changed into an average density of spikes $\varphi_{a}$, which is usually simulated by the following sigmoidal function:

1 $$\varphi_{a} = S(V_{a} ) = \frac{{2e_{0} }}{{1 + e^{{\sigma (V_{0} - V_{a} )}} }}$$

2 $$V_{a} = \sum\limits_{b} {C_{abe} } x_{b} - \sum\limits_{d} {C_{adi} } x_{d}$$

where $C_{abe}$ represents excitatory synaptic connection parameter between postsynaptic neuron population $a$ and presynaptic neuron population $b$, and $C_{adi}$ represents inhibitory synaptic connection parameter between postsynaptic neuron population $a$ and presynaptic neuron population $d$. $x_{b}$ and $x_{d}$ are postsynaptic potential (PSP) respectively generated by population $b$ and $d$. $e_{0}$ determines the maximum firing rate, $V_{0}$ is the firing threshold, $\sigma$ controls the steepness of this sigmoid function.Note that the average density of spikes for neuron population Ret is simulated by Gaussian white noise $P_{1} (t)$ with mean $\mu_{1}$ and variance $\varepsilon_{1}$. The average density of spikes for neuron population Cor is simulated by Gaussian white noise $P_{2} (t)$ with mean $\mu_{2}$ and variance $\varepsilon_{2}$.The other mathematical operation is that a second linear transform function of pulse response converts the presynaptic average spikes density $\varphi_{a}$ into the postsynaptic membrane potential $x_{a}$, which is as follows:

3 $$\tau_{a}^{2} x^{\prime\prime}_{a} = A_{a} \tau_{a} \varphi_{a} - 2\tau_{a} x^{\prime}_{a} - x_{a}$$

where $A_{a}$ is the synaptic strength determining the maximum amplitude of PSP, and $\tau_{a}$ represents the time constant of PSP. Please refer to the detailed mathematical equations governing all the 11 neuron populations in this BTC model in the Supplementary Material section.

The connectivity parameters of afferent to the brainstem and thalamus modules are according to the previous physiological data^46–50^. In detail, based on the electron microscopic observances on the connectivity patterns of two main cell types in the lateral geniculate nucleus of the cat, Erisir et al.^46^ have revealed that distribution of synapse is different between relay cells and interneurons present in this nucleus. The relative distribution of terminal types contacting relay cells is 14.6 $\pm$ 0.6% retinal (RLP) terminals, 29.6 $\pm$ 1.6% inhibitory (F) terminals, and 55.8 $\pm$ 1.7% cortex and brainstem terminals. Whereas, the analysis of the total synaptic inputs onto the interneurons indicates that interneurons receive 37.8 $\pm$ 1.0% of all synapses from retinal terminals, 26.8 $\pm$ 1.5% from inhibitory terminals, and 35.4 $\pm$ 1.8% from cortex and brainstem terminals. Moreover, in geniculate A-laminae the relay cells and the interneurons receive 85.8 $\pm$ 0.6% and 14.2 $\pm$ 0.6% of all synaptic terminals, respectively, i.e., the synaptic terminals contacting the relay cells is about 6 times of that contacting the interneurons. Erisir et al.^47^ have further found that the relative number of cortical inputs and brainstem inputs to the lateral geniculate nucleus is of the same order, each of whose terminals constitutes roughly one-half. As for the TRN afferent axons, Jones have reported that almost 70% synaptic inputs to the reticular nucleus in the somatosensory sector of the rat is attributed to corticothalamic terminals, 20–25% is attributed to thalamocortical collateral synapses, 15–20% is attributed to $GABA_{ergic}$ synapses and a quite small number of inputs is from the brainstem^48^. Another study has reported that the synaptic proportions from corticothalamic, thalamocortical and $GABA_{ergic}$ synapse are respectively 60–65%, 20% and 15%^49^. The study of Haith et al. has showed that SC and the dorsal lateral geniculate nucleus (including TRC and IN) respectively account for 15% and 85% of the retinal ganglion cells (output cells of retinal)^50^. On the basis of the above studies^46–50^ the connectivity parameters relative with the brainstem and thalamus modules are determined. On the same time, the connectivity parameters afferent to the cortex module are sourced from the works^26,28,33,34,51^. The detailed values for all the connectivity parameters are described in Table 1 in the Supplementary Material section. In addition, the synaptic strength $A_{a}$, time constant $\tau_{a}$, as well as other basic parameters ($e_{0}$, $V_{0}$, $\sigma$, $\mu_{1}$, $\varepsilon_{1}$, $\mu_{2}$, $\varepsilon_{2}$) in the BTC model are referenced from previous works^26,33,37^, which are outlined in Table 2 in the Supplementary Material section.

## Main result

As described in the Introduction, previous reports about reduced ACh release, loss of cholinergic neurons and decreased ChAT activity in AD patients have indicated that acetylcholine release is abnormal in AD patients^5–8^. In brainstem there are a large number of cholinergic neurons projecting to thalamus, moreover, the brainstem nuclei such as superior colliculus and cholinergic nucleus are susceptible to AD^9–11^. This implies that AD is associated with degeneration of the direct cholinergic pathway from brainstem to thalamus. In addition, the neuroimaging technique has shown that loss of functional/structural connectivity in cortical areas have been related to cortical atrophy in AD from graph theoretical studies^52,53^. This property of cortical atrophy may influence the descending synapse projection from cortex to brainstem^44,45^, which in turn could weaken the cholinergic pathway from brainstem to thalamus indirectly. Thus, this study simulates the pathological condition of reduced acetylcholine release in AD by decreasing the synapse connectivity parameters in the direct cholinergic pathway from brainstem to thalamus (i.e., $C_{tde}$ from PNN to TRC, $C_{idi}$ from PNN to IN, $C_{ndi}$ from PNN to TRN) and the indirect glutamatergic synapse pathway from cortex to brainstem(i.e.,$C_{dpe}$ from PY to PNN). How the abnormal acetylcholine release affects neural oscillation in this BTC model is systematically explored by means of power spectrum and amplitude distribution.

In the following, the second-order differential equations governing the BTC model are numerically solved by Euler method in an environment of MATLAB 2019a. The total simulation time is 120 s with a time resolution of 1/256 s. The model output corresponds to the summated postsynaptic potential $V_{t}$ in the TRC population. For each output vector, an epoch is extracted in the interval of [20, 120] s to ensure that all the transients are discarded. The epoch of output is then bandpass filtered by a Butterworth filter of order 10 with a lower and upper cut-off frequencies of 0.5 and 50 Hz, respectively. The detailed analysis of power spectrum and oscillation amplitude are further carried out based on the filtered output.

### Decreasing the excitatory cholinergic connectivity from PNN to TRC

The level of cholinergic neurotransmitter from the PNN population in the brainstem module to the TRC population in the thalamus module is determined by the excitatory synapse connectivity parameter $C_{tde}$. In this section, through decreasing the strength of $C_{tde}$ to mimic the degeneration of acetylcholine release in patients with AD, neural oscillation of this BTC model is firstly characterized by means of power spectrum analysis in frequency-domain. Based on the filtered output of $V_{t}$, the power spectrum density of the BTC model is computed using the Welch technique with a Hamming window. Quantitative analysis of power spectrum is performed by characterizing dominant frequency and measuring relative power in specific frequency bands. Dominant frequency is a frequency at which the power spectrum density reaches its peak. The relative power of alpha band (8-13 Hz) or theta band (4-8 Hz) is calculated by averaging the relative power spectrum density within the corresponding frequency band.

**Figure 2:**
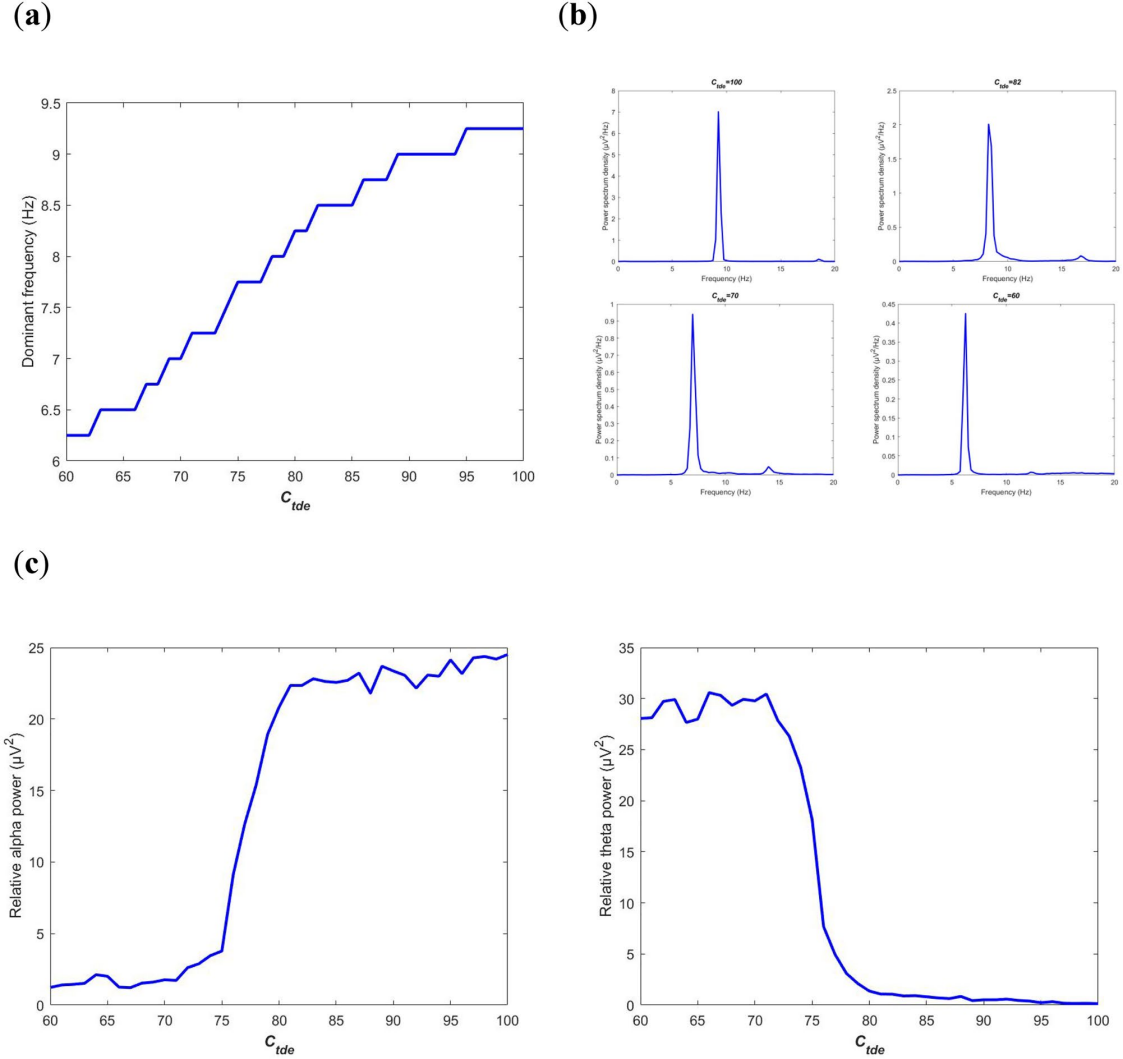
Power spectrum analysis of the BTC model output $V_{t}$ in frequency-domain when the excitatory synapse connectivity parameter $C_{tde}$ from PNN to TRC is varied in the range of [60, 100]. (**a**) Dependence of the dominant frequency of the model output on $C_{tde}$. (**b**) Individual plots of power spectrum density when $C_{tde}$ takes some different values of 100, 82, 70 and 60. (**c**) The evolution of relative power in specific frequency bands: alpha frequency band (left panel) and theta frequency band (right panel).

The dominant frequency of the model output is illustrated in Fig. 2a when the connectivity parameter $C_{tde}$ is varied in the rage of[60, 100]. On the whole, the dominant frequency shows a downward trend from 9.25 to 6.25 upon decreasing $C_{tde}$ from 100 to 60: it is initially within the alpha band, then the dominant frequency decreases steadily until $C_{tde}$ is decreased to a critical value of about 80, at which the dominant frequency transits from the alpha band to the theta band, afterwards it decreases continually within the theta band as $C_{tde}$ is further decreased. That is to say, the smaller the excitatory cholinergic synapse connectivity parameter $C_{tde}$, the lower the dominant frequency. This phenomenon is further vividly confirmed by some individual plots of power spectrum density. Figure 2b depicts the detailed power spectrum density when the connectivity parameter $C_{tde}$ is successively chosen as 100, 82, 70 and 60. Clearly, peaks of the power spectrum density in the upper two panels of Fig. 2b are concentrated at the alpha frequency band of 9.25 and 8.5 respectively when $C_{tde}$ is more than 80, whereas peaks in the lower two panels of Fig. 2b are centered at the theta frequency band of 7 and 6 respectively when $C_{tde}$ is less than 80. These interesting results indicate that acetylcholine deficiency, a biomarker for AD, can induce an obvious decrease of dominant frequency, which is consistent with the electrophysiological experiment results that the dominant/peak frequency of EEG is significantly lower in early stage of AD than that in control subjects^15–17^.

Furthermore, the power spectrum analysis of relative power in specific frequency bands is carried out. The relative power within the alpha band and within the theta band is respectively illustrated in Fig. 2c when the excitatory synapse connectivity parameter $C_{tde}$ is in the range of[60, 100]. One can observe that upon decreasing $C_{tde}$ from 100, the relative alpha band power decreases slightly until $C_{tde}$ arrives at the critical value of about 80, then it falls sharply till $C_{tde} \approx 73$, after which the relative power in alpha band does not decrease anymore and basically tends to be stable. Interestingly, the evolution of the relative theta band power is opposite to that of the relative alpha band power. In detail, upon decreasing $C_{tde}$ from 100 to 60, the relative power in theta band is just a little increased till $C_{tde}$ reaches the critical value of about 80, then it grows swiftly until $C_{tde} \approx 73$, after that the relative theta band power fluctuates slightly with the further decrease of $C_{tde}$. This phenomenon accords with the traditional experiment EEG measurements that there is a decrease in the alpha band activity and an increase in theta band activity in patients with AD^17–21^, in particular, it supports the hypothesis that cholinergic deficiency is a promising pathophysiological origin of the EEG slowing in AD^20,22^.

**Figure 3:**
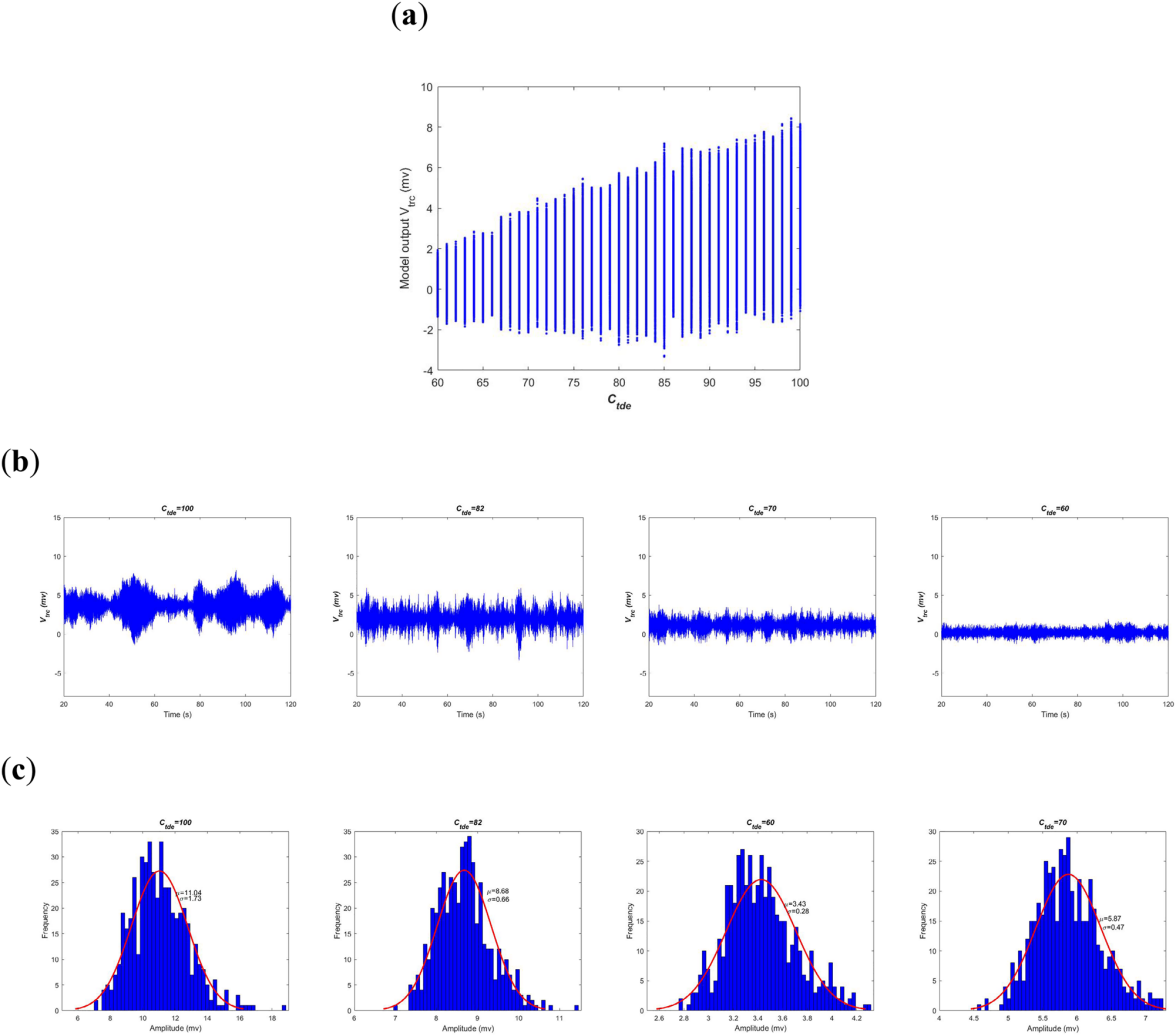
Amplitude analysis of the BTC model output $V_{t}$ in time-domain when the excitatory synapse connectivity parameter $C_{tde}$ from PNN to TRC is varied in the range of [60, 100]. (**a**) The model output of the summated postsynaptic potential $V_{t}$ for each $C_{tde}$. (**b**) Diagram of the summated postsynaptic potential $V_{t}$ during [20, 120]s when $C_{tde}$ takes some different values of 100, 82, 70 and 60. (**c**) Amplitude distribution of $V_{t}$ based on 500 realizations of postsynaptic potential when $C_{tde}$ takes some different values of 100, 82, 70 and 60.

Secondly, neural oscillation of this BTC model is investigated by means of oscillation amplitude in time-domain. When decreasing the excitatory synapse connectivity parameter $C_{tde}$ from 100 to 60, the model output, i.e., the summated postsynaptic potential $V_{t}$ in the TRC population, is illustrated in Fig. 3a for each value of $C_{tde}$. The longitudinal coordinate (y-axis) of this figure is the summated postsynaptic potential $V_{t}$ during [20, 120]s of simulations. Obviously, as a whole the oscillation amplitude of $V_{t}$ is getting smaller upon decreasing the synapse connectivity parameter $C_{tde}$. To visualize this result, the detailed postsynaptic potential $V_{t}$ for some typical synaptic strengths such as $C_{tde}$ = 100, 82, 70 and 60 is also depicted in Fig. 3b. one can qualitatively observe that the oscillation amplitude of $V_{t}$ is gradually reduced with the decrease of connectivity parameter. Especially, for the two former ones the BTC model produces rhythmic oscillation of potential analogous to alpha-rhythm (with the dominant frequency within alpha band as shown in Fig. 2b), i.e., they exhibit waxes and wanes in amplitude just like a form of spindle^32^. There is no obvious qualitative change in dynamics from the perspective of time series. As the synapse connectivity $C_{tde}$ is less than the critical value of 80, the output of BTC model in the latter two panels presents rhythmic oscillation of the theta frequency band (with the dominant frequency within the theta band as shown in Fig. 2b) with relative small amplitude compared to the former two cases. Due to the influence of the noise, statistical and uncertainty analysis is performed in order to avoid the randomness. Furthermore, as displayed in the Fig. 3c the statistical property of oscillatory potential is characterized by amplitude distribution based on 500 realizations of postsynaptic potential. Here the histogram of oscillation amplitude is fitted by normal density function with estimated parameters of mean and variance. As can be seen in the Fig. 3c, the estimated mean of oscillation amplitude is 11.04, 8.68, 5.87 and 3.43 when the connectivity parameter $C_{tde}$ is successively taken as 100, 82, 70 and 60. This result further quantitatively confirms that the reduced oscillation amplitude of the model output results from the decreased excitatory synapse connectivity parameter $C_{tde}$, i.e., a hallmark of deficit cholinergic synapse pathway from the PNN population to the TRC population.

### Decreasing the inhibitory cholinergic connectivity from PNN to IN and TRN

In addition to the above excitatory cholinergic pathway from PNN to TRC via M1 and M3 muscarinic receptor subtypes, there are also two inhibitory cholinergic pathways from PNN to IN and TRN via M2 muscarinic receptor subtype, whose synaptic connection are determined by $C_{idi}$ and $C_{ndi}$, respectively. In this section, the physiological feature of acetylcholine deficiency in AD patients is reflected by reducing $C_{idi}$ or $C_{ndi}$. Under such environment, the neural oscillation in the BTC model is investigated by means of power spectrum in frequency domain and amplitude distribution in time domain.

**Figure 4:**
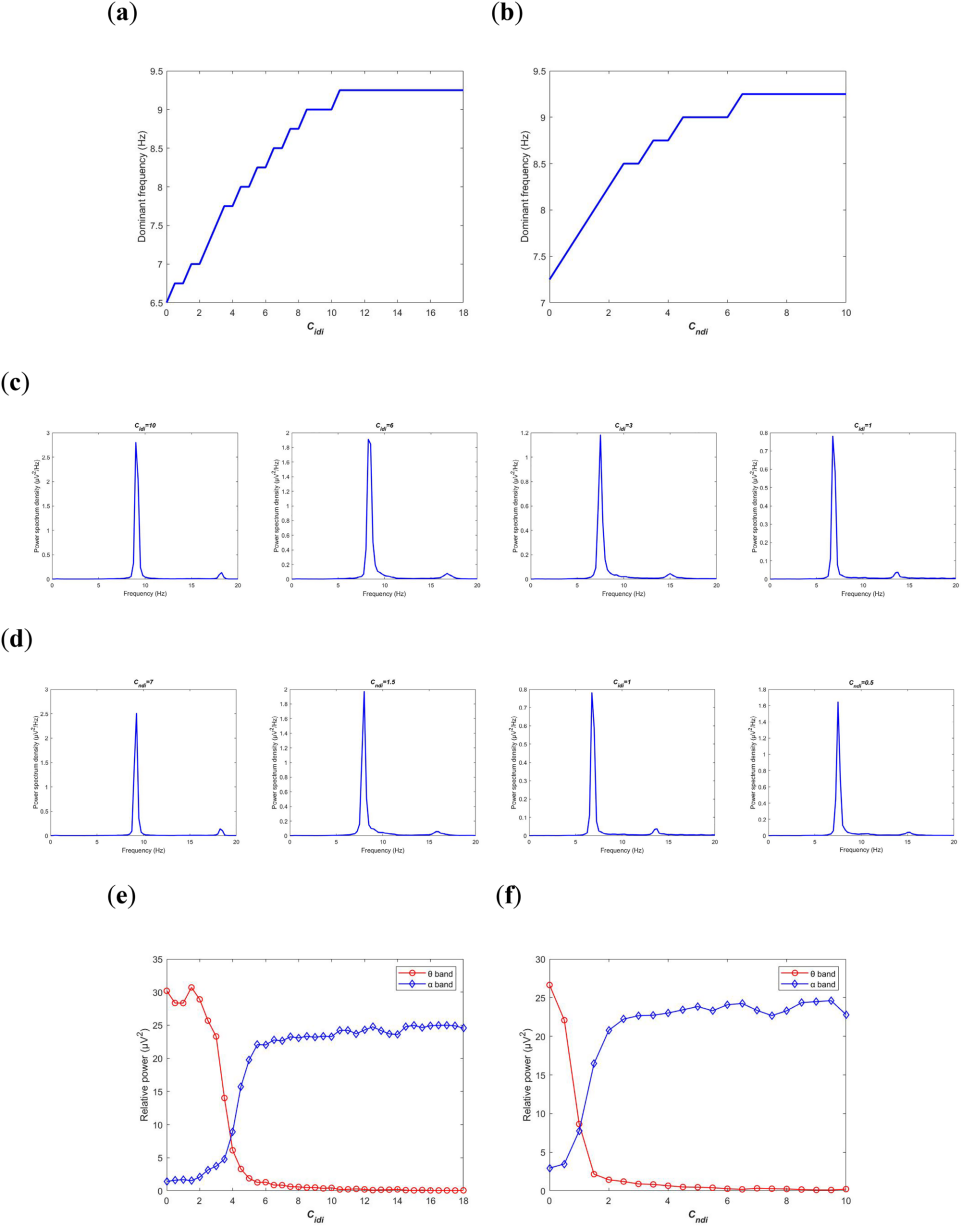
Power spectrum analysis of the BTC model output $V_{t}$ in frequency domain with decreasing of inhibitory synapse connectivity parameters $C_{idi}$ from PNN to IN and $C_{ndi}$ from PNN to TRN. Dependence of the dominant frequency of the model output on $C_{idi}$ (**a**) and $C_{ndi}$ (**b**). The individual plots of power spectrum density when $C_{idi}$ (**c**) and $C_{ndi}$ (**d**) take different values. The evolution of relative power in alpha and theta frequency bands during the reduction of $C_{idi}$ (**e**) and $C_{ndi}$ (**f**).

Firstly, neural oscillation of this BTC model is discussed on the basis of power spectrum analysis. The variation of dominant frequency of the model output $V_{t}$ with the decrease of inhibitory cholinergic synaptic strength is delineated in Fig. 4a and b. One can see that whether decreasing the synapse connectivity parameter $C_{idi}$ (from PNN to IN) or $C_{ndi}$ (from PNN to TRN), they can always lead to a decrease of dominant frequency. To be specific, when $C_{idi}$ (or $C_{ndi}$) is decreased from 18 (or 10) to a critical value of about 5 (or 2), the dominant frequency gradually drops within the alpha band, then it steps into the theta band and continually drops within theta band. To illustrate the above result more detail, some power spectrum density curves are exhibited for different synaptic connectivity parameter values. In Fig. 4c, $C_{idi}$ is taken as 10, 6, 3 and 1 from left to right. Clearly, when $C_{idi}$ is greater than 5, the frequency corresponding to the maximum of power spectrum density is respectively at 9.25 and 8.25, whereas when $C_{idi}$ is less than 5, the frequency corresponding to the maximum of power spectrum density is respectively at 7 and 6. That is to say, the dominant frequency for the left two figures and the right two figures is within the alpha band and theta band respectively. In Fig. 4d, $C_{ndi}$ is taken as 7, 3, 1.5 and 0.5 from left to right. Obviously, when $C_{ndi}$ is larger than 2, the dominant frequency in the left two panels is at the alpha frequency band of 9.25 and 8.5 respectively, while the dominant frequency in the right two panels is at the theta frequency band of 7.5 and 7.25 respectively when $C_{ndi}$ is smaller than 2. In addition, the relative power of the model output is calculated with the change of inhibitory cholinergic synapse connectivity $C_{idi}$ and $C_{ndi}$ in Fig. 4e and f. From the two panels, we can observe that upon decreasing synapse connectivity the relative theta band power (denoted by red curves with circles) increases mildly until $C_{idi}$ (or $C_{ndi}$) reaches the critical value of about 5 (or 2), then it increases sharply with the further decrease of synapse connectivity. Interestingly, the relative alpha band power (denoted by blue curves with diamonds) decreases slowly till $C_{idi}$ (or $C_{ndi}$) reaches the critical value of about 5 (or 2), after that it decreases swiftly with a further reduction of synapse connectivity.

In a word, the reduction of inhibitory cholinergic synaptic strength from the brainstem to the thalamus can change the rhythm of neural oscillation in the BTC model significantly. It can induce not only a diminished dominant frequency but also a slowing of rhythmic content, i.e., a decreased alpha band activity and an increased theta band activity. These simulated phenomena resulting from acetylcholine deficiency agree with the EEG characteristics observed in the electrophysiological experiments of AD patients, i.e., the major effect of AD on EEG is slowing of EEG^18–22^ along with the degraded peak frequency^15–17^.

**Figure 5:**
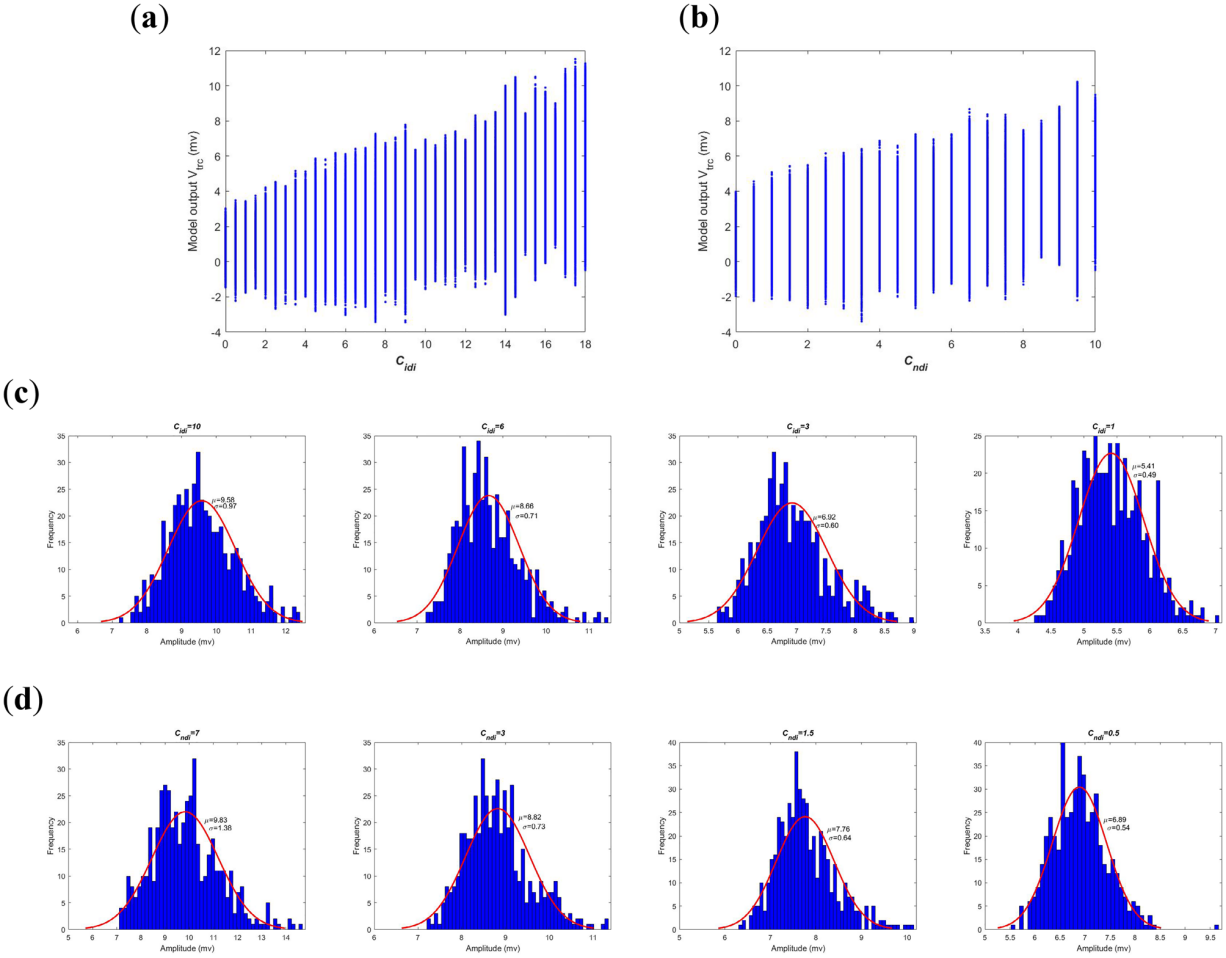
Amplitude analysis of the BTC model output $V_{t}$ in time domain with decreasing of inhibitory synapse connectivity parameters $C_{idi}$ from PNN to IN and $C_{ndi}$ from PNN to TRN. The model output of the summated postsynaptic potential $V_{t}$ for each $C_{idi}$ (**a**) and $C_{ndi}$ (**b**). Amplitude distribution of $V_{t}$ based on 500 simulation results for different $C_{idi}$ (**c**) and $C_{ndi}$ (**d**).

Secondly, the neural oscillation of the BTC model is explored in time domain by analyzing oscillation amplitude of model output. Figure 5a and b depict the variation of the summated postsynaptic potential $V_{t}$ during [20, 120]s as the inhibitory cholinergic synapse connectivity is diminished. These two figures indicate that the oscillation amplitude is degraded as a whole upon diminishing the connectivity parameter $C_{idi}$ or $C_{ndi}$, though this downward trend is not very smooth. In order to confirm this phenomenon, the detailed amplitude distribution at some synaptic strengths is further displayed in Fig. 5c and d according to the statistical property of 500 realizations of postsynaptic potential $V_{t}$. From the fitted normal density function of oscillation amplitude, the Fig. 5c reveals that the estimated mean of oscillation amplitude is successively 9.58, 8.66, 6.92 and 5.41 when $C_{idi}$ = 10, 6, 3 and 1. On the same time, the Fig. 5d reveals that the estimated mean of oscillation amplitude decreases from 9.83 to 8.82, then to 7.76, and finally to 6.89 when $C_{ndi}$ decreases from 7 to 3, then to 1.5, and finally to 0.5. These results imply that decrease of inhibitory cholinergic synapse connectivity from brainstem to thalamus resulting from acetylcholine deficit can indeed bring about a decrease in oscillation amplitude of the model output.

### Decreasing the excitatory glutamatergic connectivity from PY to PNN

As illustrated by the schematic of BTC model in **Fig. ****1**, the release of acetylcholine from brainstem to thalamus is indirectly modulated by glutamatergic synapse pathway from cortex to brainstem. The synapse loss caused by cortical atrophy in AD may destroy the descending synapse projection from cortex to brainstem^44,45^. In this section, decreasing the glutamatergic synapse connectivity $C_{dpe}$ from PY in cortex to PNN in brainstem to mimic indirect acetylcholine deficiency, neural oscillation in this BTC model is discussed using power spectrum analysis and oscillation amplitude.

**Figure 6:**
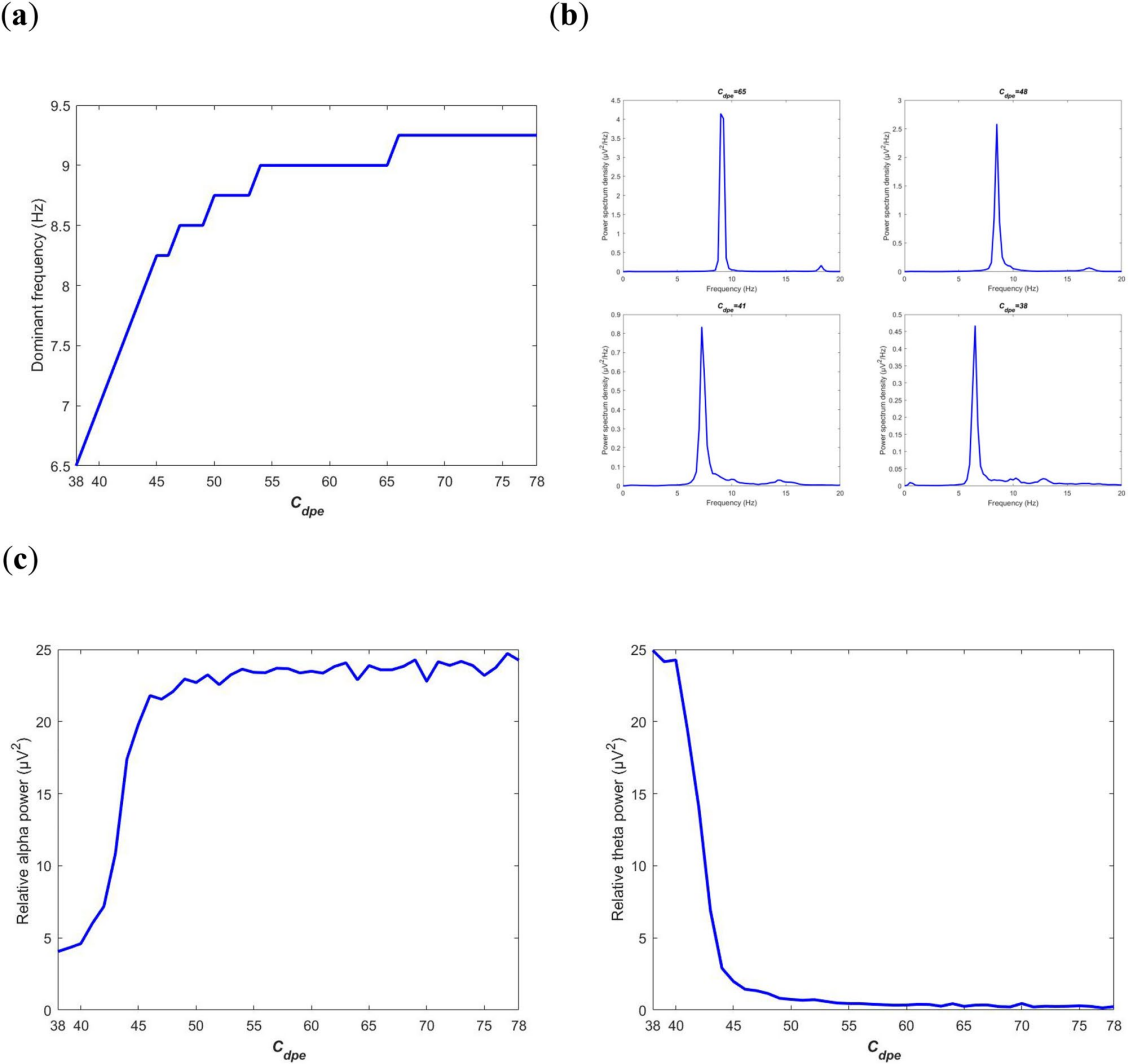
Power spectrum analysis of the BTC model output $V_{t}$ in frequency-domain when the excitatory synapse connectivity parameter $C_{dpe}$ from PY to PNN is varied in the range of[38,78]. (**a**) Dependence of the dominant frequency of the model output on $C_{dpe}$. (**b**) The power spectrum density when $C_{dpe}$ takes some different values of 65, 48, 41 and 38. (**c**) The evolution of relative power in specific bands: alpha band (left panel) and theta band (right panel).

Firstly, the property of neural oscillation is characterized by the dominant frequency and the relative power in frequency domain. The evolution of dominant frequency of the model output $V_{t}$ during the decrease of $C_{dpe}$ is depicted in Fig. 6a. Clearly, as $C_{dpe}$ is diminished from 78 to 38, the dominant frequency initially falls within the alpha frequency band until $C_{dpe}$ arrives at a critical value of 45, and then the dominant frequency continues to fall within the theta frequency band with a further decrease of $C_{dpe}$. Individual curves of power spectrum density at some synaptic connectivity are further given in Fig. 6b to confirm the above phenomenon. As shown in the upper two panels ($C_{dpe}$ = 65, 48), the peak value of power spectrum density is obtained at about 9.25 and 8.5, i.e., the dominant frequency is located within the alpha frequency band when the synaptic connectivity is large than the critical value of 45. Nevertheless, as displayed in the lower two panels ($C_{dpe}$ = 41, 38), the dominant frequency is respectively 7 and 6.5, which indicates the peak of power spectrum density is located within theta frequency band when the synaptic connectivity is smaller than the critical value of 45. Furthermore, the variation of relative power along with the synaptic connectivity $C_{dpe}$ is displayed in Fig. 6c. Upon decreasing $C_{dpe}$ from 78, the relative alpha band power in the left panel fluctuates slightly until $C_{dpe}$ reaches a certain value of 45, then it falls sharply till $C_{dpe} \approx 38$. Interestingly, an opposite phenomenon occurs for the evolution of the relative theta band power in the right panel, i.e., the relative power within theta band rises slightly until $C_{dpe}$ arrives at the certain value of 45, then it booms quickly till $C_{dpe} \approx 38$. As we expected, the rhythmic property of neural oscillation in this BTC model resulting from glutamatergic synapse deficit is similar with that what happened in the case of direct acetylcholine deficiency, i.e., the diminished dominant frequency, the degraded alpha band activity together with enhanced theta band activity, which is consistent with the EEG characteristic obtained from clinical trials of AD patients^15,17,20,22^.

**Figure 7:**
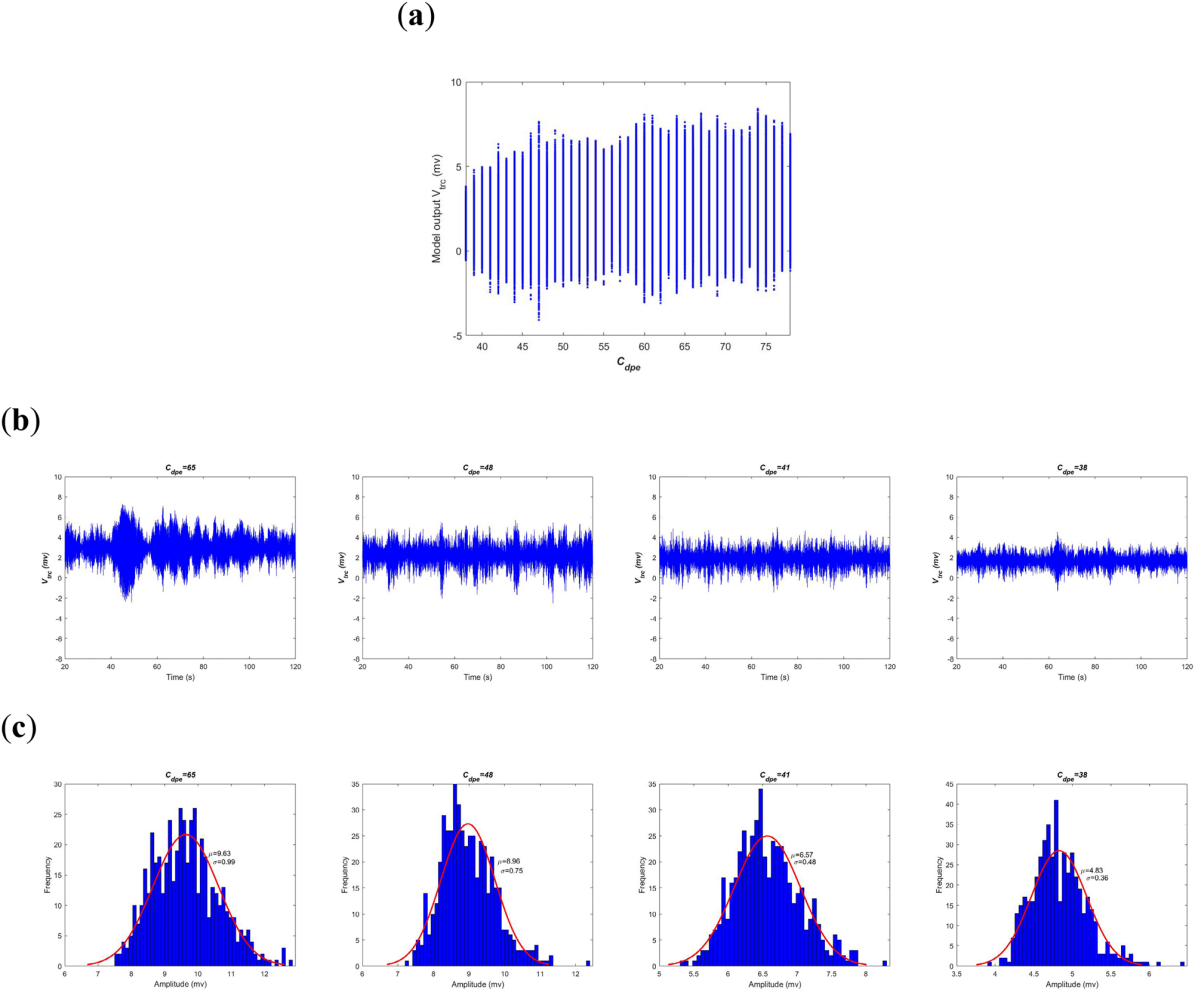
Amplitude analysis of the BTC model output $V_{t}$ in time domain when the excitatory synapse connectivity parameter $C_{dpe}$ from PY to PNN is varied in the range of[38,78]. (**a**) The model output of the summated postsynaptic potential $V_{t}$ for each $C_{dpe}$. (**b**) The summated postsynaptic potential $V_{t}$ during[20, 120]s when $C_{dpe}$ takes some values of 38, 41, 48, and 65. (**c**) Amplitude distribution of $V_{t}$ based on 500 simulation results when $C_{dpe}$ takes some values of 38, 41, 48, and 65.

Secondly, neural oscillation of model output in the BTC model is studied by means of oscillation amplitude in time domain. Figure 7a depicts the summated postsynaptic potential $V_{t}$ of model output during[20, 120]s of simulations for every connectivity parameter $C_{dpe}$ ranged in[38, 78]. Intuitively, as $C_{dpe}$ is decreased from 78 to 38 the oscillation amplitude of $V_{t}$ slowly descends as a whole except for few connectivity parameter at which the oscillation amplitude is suddenly enlarged. In addition, this result is visualized by some individual postsynaptic potential $V_{t}$ at different synaptic strength such as $C_{dpe}$ = 38, 41, 48, and 65. Combined with the dominant frequency shown in Fig. 6a, the left two panels in Fig. 7b reveal that the model output has an visible alpha rhythmic content with the amplitude waxing and waning when the synapse connectivity is greater than the critical value of 45. Whereas, in the right two panels of Fig. 7b, there appears an obvious theta rhythmic content in the postsynaptic potential with relatively small oscillation amplitude when the synapse connectivity is less than the critical value of 45. Furthermore, the corresponding amplitude distribution of 500 realizations of postsynaptic potential is illustrated in Fig. 7c. One can observe that the estimated mean of oscillation amplitude is in turn 9.63, 8.96, 6.57 and 4.83 on the basis of the fitted normal density function of oscillation amplitude. This quantitative result once again confirms that the decreased oscillation amplitude of model output is due to the damaged glutamatergic synapse connectivity $C_{dpe}$ from PY to PNN, i.e., an indirect acetylcholine synapse pathway from cortex to brainstem.

## Conclusion and discussion

This work establishes a comprehensive neural mass model in the interactive brain structures of brainstem, thalamus and cortex. It takes destroying cholinergic synapse pathway as a surrogate for the abnormal cholinergic system in patients with AD. By monitoring synapse connectivity strength in direct cholinergic pathway from brainstem to thalamus and indirect glutamatergic synapse pathway from cortex to brainstem, we are able to simulate some property of neural oscillation in this model under an environment of acetylcholine deficiency from the standpoint of neurocomputation. By analyzing power spectrum of the model output in frequency domain, the results reveal that upon diminishing synapse connectivity strength in a certain range the dominant frequency initially decreases within alpha frequency band and then steps into theta frequency band, which is consistent with the electrophysiological experiment results that the dominant/peak frequency of EEG is significantly lower in early stage of AD than that in control subjects^15–17^. Meanwhile, the neural oscillation presents a slowing rhythmic content with a degraded alpha band relative power and an enhanced theta band relative power, which accords with the traditional experiment EEG measurements that there is a decrease in the alpha band activity and an increase in the theta band activity in patients with AD^17–21^. In particular, it supports the hypothesis that cholinergic deficiency is a promising pathophysiological origin of the EEG slowing in AD^20,22^. What’s more, amplitude distribution of the neural oscillation in time domain suggests that the oscillation amplitude is overall diminished with the reduction of synapse connectivity. We expect these findings could have important implications on better understanding cholinergic pathogenesis and expounding potential feature for AD.

At last, we point out that this work, by reducing the synaptic connection parameters in cholinergic direct pathway from brainstem to thalamus or indirect glutamatergic synapse pathway from cortex to brainstem, mainly focus on the effect of acetylcholine deficiency on brain neuron activity in patients with AD. As known that there could be neuron loss and brain atrophy in cortical region in AD patients^44,45^, which would induce unfavorable synapse information processing. Thus, reducing the synaptic connection parameters relative to cortical region can also simulate the pathological state of AD. Through further simulation, we find that when synapse connectivity parameter decreases from PY to TRC, the change of the dominant frequency and the oscillation amplitude is similar with what reported in this work, i. e. there is an obvious decrease of dominant frequency, a degraded rhythmic activity in the alpha frequency band as well as an enhanced rhythmic activity in the theta frequency band, and the reduced oscillation amplitude of the model output. For the limited space, we do not list them in detail.

## Supplementary Information

Supplementary Information.

## Data Availability

All data generated or analyzed during this study are included in this published article.
