# Supplementary material for: Effect of acetylcholine deficiency on neural oscillation in a brainstem-thalamus-cortex neurocomputational model related with Alzheimer’s disease
Source: Sci Rep. 2022 Sep 2;12:14961. doi: 10.1038/s41598-022-19304-3 (PMC9440203; doi:10.1038/s41598-022-19304-3)
Supplement: Supplementary file 1 — Supplementary Information. [file 41598_2022_19304_MOESM1_ESM.docx]

**Nature Scientific Report**

**Supplementary Material**

**Effect of acetylcholine deficiency on neural oscillation in a brainstem-thalamus-cortex neurocomputational model related with Alzheimer's disease**

Hao Yang^1^, XiaoLi Yang[[1]](#footnote-0)*1, SiLu Yan^1^, ZhongKui Sun^2^

^1^School of Mathematics and Statistics, Shaanxi Normal University, Xi’an 710062,

 PR China

^2^ School of Mathematics and Statistics, Northwestern Polytechnical University, Xi’an

710072, PR China

(*^*^* Corresponding author: XiaoLi Yang, yangxiaoli@snnu.edu.cn )

1. **The detailed mathematical equations for the BTC model**

The layout for module structure and synaptic connectivity in the BTC model is illustrated in Figure 1, which has 11 interconnected neuronal populations (SC, PNN, IN, TRC, TRN, eIN, PY, fIN, sIN, Ret, Cor). As described in Section 2, the average membrane potential $V_{a}$ in each population receiving postsynaptic membrane potential from all afferent neuronal populations is changed into an average density of spikes $\phi_{a}$(Eqs. (1-2)), then the presynaptic spike density $\phi_{a}$ is converted into the postsynaptic membrane potential $x_{a}$ by a second linear transform function of pulse response (Eq. (3)). The detailed mathematical equations governing all the 11 neuronal populations reads as follows:

**Brainstem Module**

$$V_{d}=C_{dpe}x_{p}$$

$$V_{s}=C_{sre}x_{r}+C_{sde}x_{d}+C_{spe}x_{p}$$

**Thalamic Module**

$V_{t}=C_{tre}x_{r}+C_{tpe}x_{p}+C_{tse}x_{s}+C_{tde}x_{d}-C_{tii}x_{i}-C_{tni}x_{n}$

$$V_{i}=C_{ire}x_{r}+C_{ipe}x_{p}+C_{ise}x_{s}-C_{iii}x_{i}-C_{idi}x_{d}$$

$$V_{n}=C_{nte}x_{t}+C_{npe}x_{p}-C_{nni}x_{n}-C_{ndi}x_{d}$$

**Cortical Module**

$$V_{p}=C_{pce}x_{c}+C_{pte}x_{t}+C_{phe}x_{h}-C_{pli}x_{l}-C_{pfi}x_{fin}$$

$$V_{h}=C_{hpe}x_{p}+C_{hte}x_{t}$$

$$V_{l}=C_{lpe}x_{p}+C_{lte}x_{t}-C_{lfi}x_{f}$$

$$V_{f}=C_{fpe}x_{p}+C_{fte}x_{t}-C_{fli}x_{l}$$

**Extrinsic Source**

1. **Model Parameters**

As Stated out in Section 2, the connectivity parameters relative with the brainstem and thalamus modules are determined on the basis of previous studies [46-50]. The connectivity parameters afferent to the cortex module are sourced from the works [26, 28, 33, 34, 51]. The detailed values for all the connectivity parameters in the BTC model are described in the following Table 1. In addition, the synaptic strength $A_{a}$, time constant$\tau_{a}$, as well as other parameters ($e_{0}$, $V_{0}$, $\sigma$, $\mu_{1}$, $\varepsilon_{1}$, $\mu_{2}$, $\varepsilon_{2}$) in the BTC model are referenced from previous works [33, 26, 37], which are outlined in the following Table 2.

**Table 1** Parameters of synaptic connectivity between different neuron populations in BTC model.

| Symbol | Postsynaptic neuron population | Presynaptic neuron population | Value | Symbol | Postsynaptic neuron population | Presynaptic neuron population | Value |
| --- | --- | --- | --- | --- | --- | --- | --- |
| $C_{sre}$ | SC | Ret | 8.5 | $C_{pce}$ | PY | Cor | 1 |
| $C_{sde}$ |  | PNN | 8 | $C_{pte}$ |  | TCR | 80 |
| $C_{spe}$ |  | PY | 20 | $C_{phe}$ |  | eIN | 108 |
| $C_{dpe}$ | PNN | PY | 60 | $C_{pli}$ |  | sIN | 33.75 |
| $C_{tre}$ | TCR | Ret | 48.2 | $C_{pfi}$ |  | fIN | 108 |
| $C_{tii}$ |  | IN | 48.8 | $C_{hte}$ | eIN | TCR | 100 |
| $C_{tni}$ |  | TRN | 48.8 | $C_{hpe}$ |  | PY | 135 |
| $C_{tpe}$ |  | PY | 92 | $C_{lte}$ | sIN | TCR | 40 |
| $C_{tde}$ |  | PNN | 92 | $C_{lpe}$ |  | PY | 33.75 |
| $C_{tse}$ |  | SC | 50 | $C_{lfi}$ |  | fIN | 13.5 |
| $C_{ire}$ | IN | Ret | 20.8 | $C_{fte}$ | fIN | TCR | 40 |
| $C_{isi}$ |  | IN | 14.7 | $C_{fpe}$ |  | PY | 40.5 |
| $C_{ipe}$ |  | PY | 9.7 | $C_{fli}$ |  | sIN | 13.5 |
| $C_{ise}$ |  | SC | 30 | $C_{nsi}$ | TRN | TRN | 8.3 |
| $C_{idi}$ |  | PNN | 9.7 | $C_{npe}$ |  | PY | 30.3 |
| $C_{nte}$ | TRN | TCR | 11 | $C_{ndi}$ |  | PNN | 5.5 |

**Table2** Values of synaptic strength, time constant, and other basic parameters in BTC model.

| Symbol | Unit | Value | Symbol | Unit | Value | Symbol | Unit | Value |
| --- | --- | --- | --- | --- | --- | --- | --- | --- |
| $A_{d}$ | $mv$ | 5.2 | $\tau_{d}$ | $s$ | 1/160 | $e_{0}$ | $s^{-1}$ | 2.5 |
| $A_{s}$ |  | 2.4 | $\tau_{s}$ |  | 1/80 | $V_{0}$ | $mv$ | 6 |
| $A_{t}$ |  | 3.25 | $\tau_{t}$ |  | 1/100 | $\sigma$ | $mv^{-1}$ | 0.56 |
| $A_{i}$ |  | 22 | $\tau_{i}$ |  | 1/40 | $\mu_{1}$ | $sps$ | 11 |
| $A_{n}$ |  | 22 | $\tau_{n}$ |  | 1/40 | $\varepsilon_{1}$ | $sps$ | 2 |
| $A_{p}$ |  | 2.7 | $\tau_{p}$ |  | 1/40 | $\mu_{2}$ | $sps$ | 30 |
| $A_{x}$ |  | 2.7 | $\tau_{x}$ |  | 1/40 | $\varepsilon_{2}$ | $sps$ | 2 |
| $A_{f}$ |  | 29 | $\tau_{f}$ |  | 1/300 | $sps$(spikes per second) | | |
| $A_{l}$ |  | 4.5 | $\tau_{l}$ |  | 1/20 |  |  |  |
| $A_{r}$ |  | 3.25 | $\tau_{r}$ |  | 1/100 |  |  |  |
| $A_{c}$ |  | 2.7 | $\tau_{c}$ |  | 1/40 |  |  |  |

1. [↑](#footnote-ref-0)
